# Aetiology of community-acquired pneumonia in the ICU setting and its effect on mortality, length of mechanical ventilation and length of ICU stay: a 1-year retrospective review

**DOI:** 10.1186/cc14067

**Published:** 2014-12-03

**Authors:** D Ryan, R Connolly, J Fennell, G Fitzpatrick

**Affiliations:** 1 The Adelaide and Meath Hospital, incorporating the National Children's Hospital (AMNCH), Tallaght, Dublin, Ireland

## Introduction

Community-acquired pneumonia (CAP) is the most common infectious reason for admission to ICUs and has mortality of up to 37% [1]. Highest mortality rates are in Gram-negative infections with lower rates in *Streptococcus pneumoniae *and viral infections [2]. Microbiology is difficult to establish with most prospective studies identifying agents in only 50% of cases [3]. We analysed microbial aetiology of CAP in ICU over 1 year and assessed its effects on inpatient mortality, length of mechanical ventilation and length of ICU stay.

## Methods

We retrospectively reviewed admissions to AMNCH ICU between February 2013 and February 2014 catalogued as having pneumonia from chest radiograph and clinical findings on the internal audit system (*n *= 91). After chart review, 28 were excluded as hospital-acquired pneumonias, 12 due to insufficient information and 21 due to primary diagnosis other than pneumonia. Thirty patients were selected on the basis that CAP was the likely reason for ICU admission.

## Results

Pathogens were detected in 73% of patients by culture, antigen detection or molecular methods: *S. pneumoniae *(*n *= 6; 20%), influenza (*n *= 4; 13%), viral with superimposed bacterial infection (*n *= 4; 13%), viruses other than influenza (*n *= 3; 10%), Gram-negative bacilli (*n *= 2; 7%), *Legionella *spp. (*n *= 2; 7%) and *Haemophilus *spp. (*n *= 1; 3%). No organism was identified in 27%. Gram-negative infection had highest mortality (100%), average length of mechanical ventilation (57 days) and average ICU stay (64 days). The mean age of affected patients was 68.2. Mortality was lower in influenza/other viral infections (75/50%), patients spent less time mechanically ventilated (mean 6.6/13.3 days) and less time in ICU (mean 4/16.3 days), reflecting the younger mean age of patients (53.2/51.4). Of patients infected with *S. pneumonia*e, *Legionella *spp. or *Haemophilus *spp. alone, all survived to discharge. However, of note, when superimposed on viral infection, these pathogens carried a higher mortality (50%). Infection with pneumococcus alone occurred in younger patients (mean age 50.6) and was associated with a shorter ventilation period (mean 2.66 days) and ICU stay (mean 6 days).

## Conclusion

Microbial aetiology was identified in a high proportion of patients (72%) admitted to ICU with CAP, reflecting timely collection of appropriate specimens. Infection with Gram-negative organisms had the highest mortality, length of mechanical ventilation and length of ICU stay, while the pathogens usually seen in CAP were associated with more favourable outcomes.
